# Development and validation of a nomogram for prediction of cervical lymph node metastasis in middle and lower thoracic esophageal squamous cell carcinoma

**DOI:** 10.1186/s12876-022-02243-8

**Published:** 2022-04-03

**Authors:** Zhaoyang Yan, Xinjian Xu, Juntao Lu, Yang You, Jinsheng Xu, Tongxin Xu

**Affiliations:** 1 Department of Thoracic Surgery, The Fourth Hospital of Hebei Medical University, Shijiazhuang, Hebei Province China; 2 Laboratory of Pathology, Hebei Cancer Institute, The Fourth Hospital of Hebei Medical University, Shijiazhuang, Hebei Province China; 3 Department of CT&MRI, The Fourth Hospital of Hebei Medical University, Jiankang Rd. 12, Shijiazhuang, 050011 Hebei Province China; 4 Hebei Key Laboratory of Vascular Calcification in Kidney Disease, Hebei Clinical Research Center for Chronic Kidney Disease, The Fourth Hospital of Hebei Medical University, Shijiazhuang, Hebei Province China

**Keywords:** Cervical lymph node metastasis, Esophageal squamous cell carcinoma, Nomogram

## Abstract

**Background:**

Estimates of cervical lymph node (LN) metastasis in patients with middle and lower thoracic esophageal squamous cell carcinoma (ESCC) are important. A nomogram is a useful tool for individualized prediction.

**Methods:**

A total of 235 patients were enrolled in this study. Univariate and multivariate analyses were performed to screen for independent risk factors and construct a nomogram to predict the risk of cervical LN metastasis. The nomogram performance was assessed by discrimination, calibration, and clinical use.

**Results:**

Totally, four independent predictors, including the maximum diameter of tumor, paraesophageal lymph node status, recurrent laryngeal nerve lymph node status, and the CT-reported cervical LN status, were enrolled in the nomogram. The AUC of the nomogram model in the training and validation dataset were 0.833 (95% CI 0.762–0.905), 0.808 (95% CI 0.696–0.920), respectively. The calibration curve demonstrated a strong consistency between nomogram and clinical findings in predicting cervical LN metastasis. Decision curve analysis demonstrated that the nomogram was clinically useful.

**Conclusion:**

We developed a nomogram that could be conveniently used to predict the individualized risk of cervical LN metastasis in patients with middle and lower thoracic ESCC.

**Supplementary Information:**

The online version contains supplementary material available at 10.1186/s12876-022-02243-8.

## Introduction

Esophageal cancer, including esophageal squamous cell carcinoma (ESCC) and esophageal adenocarcinoma (EAC), ranks the eighth in the most common cancer in the world [1]. It was reported that the 5-year survival rates ranged from 20 to 35% in the nonmetastatic setting [2]. ESCC is the predominant type in China and this malignant tumor is associated with high morbidity and mortality as well as clear geographical heterogeneity [3]. Currently, definitive chemoradiotherapy has been recognized as a standard treatment for locally advanced unresectable ESCC. Surgery is still the mainstay of treatment for resectable ESCC, although neoadjuvant therapy is increasingly being accepted [4].

Lymphadenectomy is an essential part of the surgical treatment of ESCC, but the dissection extent of lymph node (LN) remains controversial, especially when the tumor is located in the middle and lower esophagus [5, 6]. Three-field (cervical-thoracic-abdominal) lymphadenectomy adds the excision of cervical LNs on the basis of two-field lymphadenectomy. Extended lymphadenectomy could remove more potentially metastatic LNs, provide accurate tumor staging, guide appropriate postoperative adjuvant therapy and become a useful predictive tool for prognosis prediction. But this notion was challenged by some clinical randomized controlled trials [7, 8], which suggested that ESCC patients may not benefit from the dissection of the cervical LNs. Furthermore, three-field lymphadenectomy may also increase the complexity of the operation and the incidence of some complications, such as blood loss, anastomotic fistula, and recurrent nerve palsy [9, 10]. Therefore, prediction of cervical LN metastasis could provide an individualized LN dissection way and a reference for choosing optimal surgical procedures.

The present study aimed to develop a nomogram that contained clinicopathologic risk factors for individual prediction of cervical LN metastasis in patients with middle and lower thoracic ESCC to help clinicians develop better treatment protocols.

## Materials and methods

### Patients

**Fig. 1:**
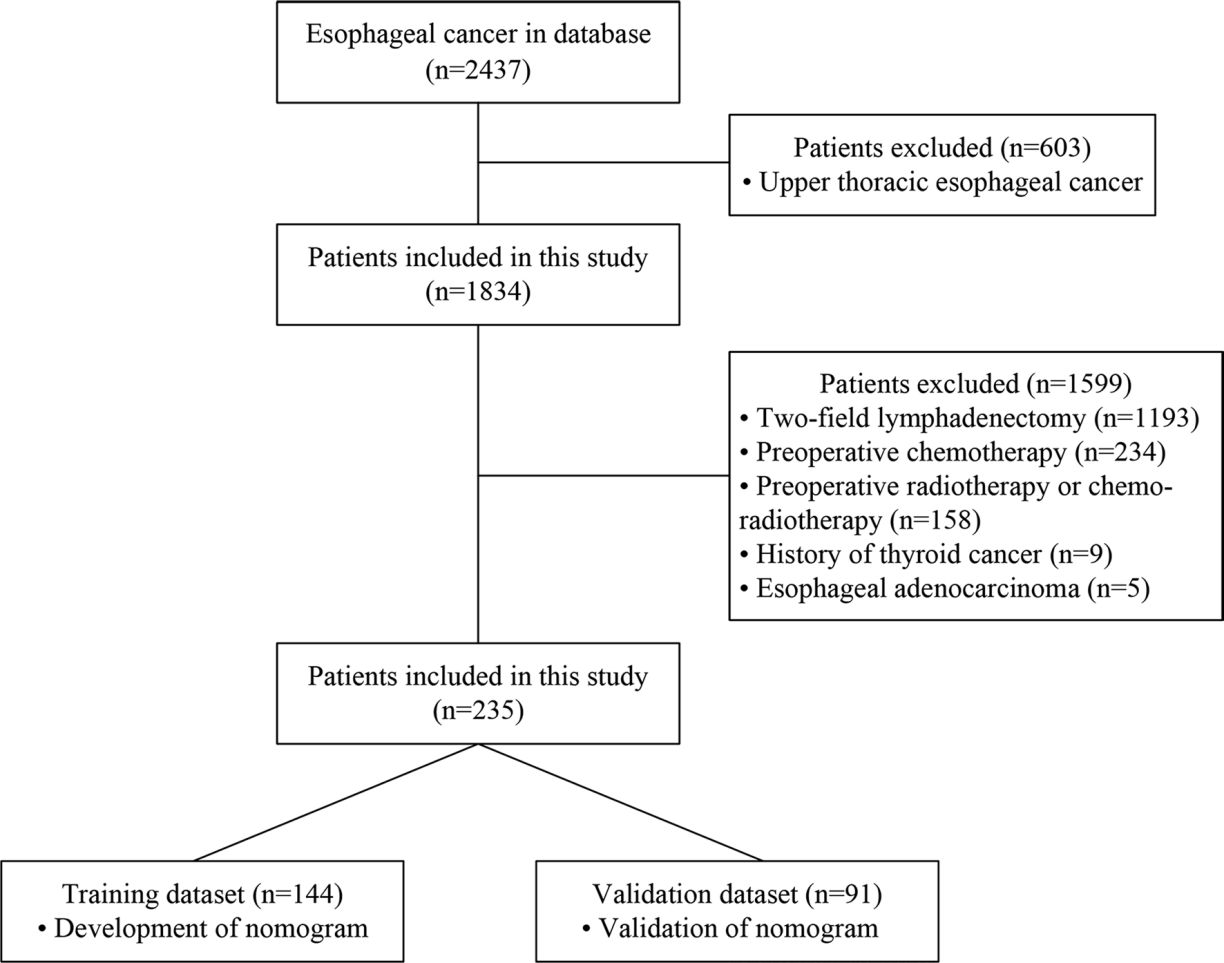
Flow diagram of patient enrollment and study design

Between January 2015 and December 2019, 506 patients with esophageal cancer underwent radical esophagectomy with three-field lymphadenectomy at the Fourth Hospital of Hebei Medical University. Among these patients, the following were excluded from the study population: 172 were diagnosed as upper thoracic esophageal cancer; 59 with preoperative chemotherapy; 34 with preoperative radiotherapy or chemo-radiotherapy; 4 with a history of thyroid cancer; 2 were diagnosed as EAC. Finally, a total of 235 patients were enrolled in this study. We then randomly divided the patients into a training dataset (n = 114) and a validation dataset (n = 91) at a ratio of 6:4 (Fig. 1). This study was reviewed and approved by the Ethics Committee of the Fourth Hospital of Hebei Medical University (2021KY138). The requirement to obtain informed consent was waived owing to the retrospective design of the study.

### Clinicopathologic characteristics

General information of the patients, including gender and age, were recorded. Preoperative gastroscopy and standard contrast-enhanced CT were performed to determine the tumor location and maximum diameter of the tumor. The tumor location of the upper, middle, and lower was diagnosed when the upper end of the tumor was located 15–25, 25–30, or 30–45 cm from an upper incisor, respectively [11]. Considering that small tumors such as the T1 stage of ESCC may be difficult to detect in CT images, the gastroscopy was alone used to define the maximum diameter of tumor when CT was not available to detect the lesion. The maximum diameter of tumor was recorded with continuous variables.

For the cervical lymphadenectomy, all the LNs in the lower neck were resected, including cervical paraesophageal LNs, deep cervical LNs, and supraclavicular LNs. The detailed location of cervical LNs was shown in Additional file 2: Fig. S1. The thoracic LNs stations were classified according to the Chinese expert consensus on mediastinal lymph node dissection in esophagectomy for esophageal cancer (2017 edition) [12]. As shown in Additional file 3: Fig. S2, Station C201 and C202 were defined as recurrent laryngeal nerve lymph node (RLN LN); Station C203, C206, and C207 were defined as paraesophageal lymph node (PLN); Station C204, C205, C208, and C209 were defined as mediastinal lymph node (MLN). The abdominal lymph node (ALN) included the paracardial, lesser curvature, greater curvature, left gastric artery, and hepatic artery LNs. Surgically dissected LNs were labeled for pathological examination according to the definition of regional LNs.

The criteria of CT for the diagnosis of cervical LN metastasis were defined as follow: (1) target LNs with a long axis of ≥ 10 mm or a short axis of ≥ 5 mm [13]; (2) LNs with a short-to-long axis ratio of > 0.5 [14]; or (3) presence of internal features such as necrosis, cystic lesion, and extracapsular spread according to the previous report [15]. The CT-reported cervical LN status was confirmed by two professional radiologists. The depth of tumor invasion, PLN status, MLN status, RLN LN status, ALN status, blood vessel invasion, nerve invasion, and cervical LN status were obtained from the final pathology reports completed by two professional pathologists.

### Statistical analysis

The baseline characteristics are summarized as median (interquartile range [IQR]) for continuous variables and frequency (percentages) for categorical variables. Wilcoxon rank sum test was applied to compare continuous variables and chi-square test or Fisher’s exact test was applied to analyze categorical variables. Forward stepwise selection with an inclusion criterion of a *P* < 0.1 was performed to construct the final multivariable prediction model. For further analysis, a nomogram was formulated based on the results of multivariable logistic regression analysis. The discrimination of the nomogram was assessed using the receiver operating characteristic (ROC) curves with area under the ROC curve (AUC). The calibration curve was evaluated by unreliability U test. Decision curve analysis was used to determine the clinical usefulness of the nomogram by quantifying the net benefits at different threshold probabilities in the validation dataset [16]. All of the data were analyzed using the STATA 15.0 (StataCorp Texas, USA) and R software (version 3.5.2; http://www.Rproject.org). The reported statistical significance levels were all two-sided, with statistical significance set at 0.05.

## Results

### Clinicopathological features of patients

The study population included 235 middle and lower ESCC patients. The clinicopathological characteristics of patients in both the training and validation datasets are given in Table 1. There were no statistically significant differences in all variables between the two datasets. Cervical lymph node metastasis positivity was 25.0% and 25.3% in the training and validation datasets, respectively.

**Table 1:** Characteristics of patients in the training and validation dataset

Characteristic	Training dataset			Validation dataset			*P*
	Total	Cervical LN Metastasis		Total	Cervical LN Metastasis		
		(−) (n = 108)	(+) (n = 36)		(−) (n = 68)	(+) (n = 23)	
*Gender, No. (%)*							0.26
Male	100 (69.4)	75 (69.4)	25 (69.4)	56 (61.6)	38 (55.9)	18 (78.3)	
Female	44 (30.6)	33 (30.6)	11 (30.6)	35 (38.5)	30 (44.1)	5 (22.7)	
Age, median (IQR), years	62 (58.5, 65.5)	62.5 (58.5, 66)	61.5 (58, 64.5)	62 (58, 67)	62.5 (58.5, 67)	60 (54, 65)	0.69
*Tumor location, No. (%)*							0.75
Middle	111 (77.1)	80 (74.1)	31 (86.1)	68 (74.7)	52 (76.5)	16 (69.6)	
Lower	33 (22.9)	28 (25.9)	5 (13.9)	23 (25.3)	16 (23.5)	7 (30.4)	
*Degree of differentiation, No. (%)*							0.44
Well	18 (12.5)	14 (13.0)	4 (11.1)	17 (18.7)	16 (23.5)	1 (4.3)	
Moderate	85 (59.0)	62 (57.4)	23 (63.9)	51 (56.0)	37 (54.4)	14 (60.9)	
Poorly	41 (28.5)	32 (29.6)	9 (25.0)	23 (25.3)	15 (22.1)	8 (34.8)	
Maximum diameter of tumor, median (IQR), cm	3.5 (2.5, 5)	3.5 (2.45,4.5)	4.25 (3, 5.75)	3 (2, 4)	3 (2, 4)	4 (3, 5)	0.12
*Depth of tumor invasion, No. (%)*							0.37
T1	32 (22.2)	29 (26.9)	3 (8.3)	28 (30.8)	27 (39.7)	1 (4.3)	
T2	23 (16.0)	18 (16.7)	5 (13.9)	17 (18.7)	15 (22.1)	2 (8.7)	
T3	71 (49.3)	50 (46.3)	21 (58.3)	38 (41.8)	22 (32.4)	16 (69.6)	
T4	18 (12.5)	11 (10.2)	7 (19.4)	8 (8.8)	4 (5.9)	4 (17.4)	
*PLN status No. (%)*							0.15
Negative	115 (79.9)	94 (87.0)	21 (58.3)	80 (87.9)	64 (94.1)	16 (69.6)	
Positive	29 (20.1)	14 (13.0)	15 (41.7)	11 (12.1)	4 (5.9)	7 (30.4)	
*MLN status No. (%)*							0.85
Negative	122 (84.7)	97 (89.8)	25 (69.4)	76 (83.5)	64 (94.1)	12 (52.2)	
Positive	22 (15.3)	11 (10.2)	11 (30.6)	15 (16.5)	4 (5.9)	11 (47.8)	
*RLN LN status No. (%)*							0.48
Negative	93 (64.6)	80 (74.1)	13 (36.1)	63 (69.2)	55 (80.9)	8 (34.8)	
Positive	51 (35.4)	28 (25.9)	23 (63.9)	28 (30.8)	13 (19.1)	15 (65.2)	
*ALN status No. (%)*							0.56
Negative	99 (68.8)	75 (69.4)	24 (66.7)	66 (72.5)	56 (82.4)	10 (43.5)	
Positive	45 (31.3)	33 (30.6)	12 (33.3)	25 (27.5)	12 (17.6)	13 (56.5)	
*Blood vessel invasion, No. (%)*							0.84
Negative	128 (88.9)	99 (91.7)	29 (80.6)	80 (87.9)	64 (94.1)	16 (69.6)	
Positive	16 (11.1)	9 (8.3)	7 (19.4)	11 (12.1)	4 (5.9)	7 (30.4)	
*Nerve invasion, No. (%)*							0.86
Negative	121 (84.0)	93 (86.1)	28 (77.8)	75 (82.4)	59 (86.8)	16 (69.6)	
Positive	23 (16.0)	15 (13.9)	8 (22.2)	16 (17.6)	9 (13.2)	7 (30.4)	
*CT-reported cervical LN status, No. (%)*							0.63
Negative	113 (78.5)	91 (84.3)	22 (61.1)	69 (75.8)	55 (80.9)	14 (60.9)	
Positive	31 (21.5)	17 (15.7)	14 (38.9)	22 (24.2)	13 (19.1)	9 (39.1)	

*LN* lymph node, *PLN* paraoesophageal lymph node, *MLN* mediastinal lymph node, *RLN LN* recurrent laryngeal nerve lymph node, *ALN* abdominal lymph node

In the training dataset, 77.1% of the tumors were located in the middle esophagus and 22.9% were located in the lower esophagus. In histology differentiation, the ratios of well, moderate, and poorly grade were 12.5%, 59.0%, and 28.5%, respectively. In depth of tumor invasion, the ratios of T1, T2, T3, and T4 were 22.2%, 16.0%, 49.3%, and 12.5%, respectively. The median and IQR (Q1–Q3) maximum diameter of tumor was 3.5 cm (2.5, 5 cm). The ratios of PLN status (+), MLN status (+), RLN LN status (+), ALN status (+), Blood vessel invasion (+), Nerve invasion (+), and CT-reported cervical LN status (+) were 20.1%, 15.3%, 35.4%, 31.3%, 11.1%, 16.0%, and 21.5%, respectively.

### Predictors of cervical LN metastasis

To identify the predictive factors of cervical LN metastasis, univariate and multivariate logistic regression analysis were performed in the training cohort (Table 2). The univariate analysis showed that risk factors for cervical LN metastasis included maximum diameter of tumor, PLN status, MLN status, RLN LN status, and CT-reported cervical lymph node status. In multivariate analysis, four variables, which included maximum diameter of tumor (OR 1.46, 95% CI 1.11–1.92), PLN status (OR 3.94, 95% CI 1.45–10.68), RLN LN status (OR 3.82, 95% CI 1.56–9.34) and CT-reported cervical lymph node status (OR 4.43, 95% CI 1.60–12.27) were proved to be independent risk factors for cervical LN metastasis.

**Table 2:** Predictive factors for cervical LN metastasis (144 cases)

Predictors	Univariate analysis		Multivariable analysis	
	*P*	OR (95% CI)	*P*	OR (95% CI)
Gender	1.00		–	
Male		Reference		–
Female		1.00 (0.44–2.27)		–
Age, years	0.47	0.99 (0.94–1.05)	–	–
Tumor location	0.17		–	
Middle		Reference		–
Lower		0.46 (0.16–1.30)		–
Degree of differentiation	0.82		–	
Well		Reference		–
Moderate		1.29 (0.38–4.35)		–
Poorly		0.98 (0.26–3.74)		–
Maximum diameter of tumor	< 0.01	1.43 (1.14–1.80)	< 0.01	1.46 (1.11–1.92)
Depth of tumor invasion	0.06		–	
T1		0.37 (0.08–1.75)		–
T2		Reference		–
T3		1.51 (0.50–4.61)		–
T4		2.29 (0.58–9.02)		–
PLN status	< 0.01		< 0.01	
Negative		Reference		Reference
Positive		4.80 (2.01–11.43)		3.94 (1.45–10.68)
MLN status	< 0.01		–	
Negative		Reference		–
Positive		3.88 (1.51–9.98)		–
RLN LN status	< 0.01		< 0.01	
Negative		Reference		Reference
Positive		5.05 (2.26–11.30)		3.82 (1.56–9.34)
ALN status	0.84		–	
Negative		Reference		–
Positive		1.14 (0.51–2.54)		–
Blood vessel invasion	0.12		–	
Negative		Reference		–
Positive		2.66 (0.91–7.75)		–
Nerve invasion	0.29		–	
Negative		Reference		–
Positive		1.77 (0.68–4.61)		–
CT-reported cervical LN status	< 0.01		< 0.01	
Negative		Reference		Reference
Positive		3.41 (1.46–7.94)		4.43 (1.60–12.27)

*OR* odds ratio, *CI* confidence interval

### Development and validation of the nomogram for the prediction of cervical LN metastasis

**Fig. 2:**
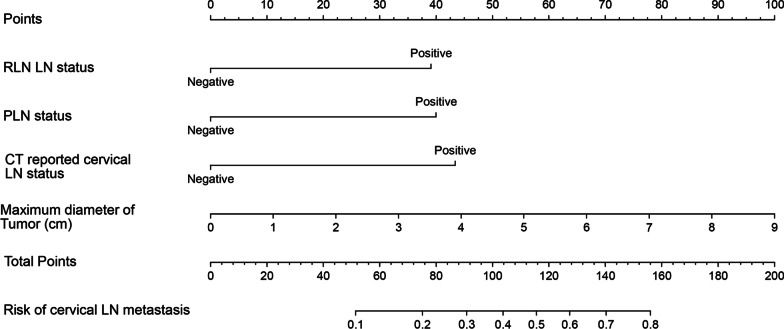
Nomogram for prediction of cervical lymph node metastasis. LN: lymph node; PLN: paraesophageal lymph node; RLN LN: recurrent laryngeal nerve lymph node

**Fig. 3:**
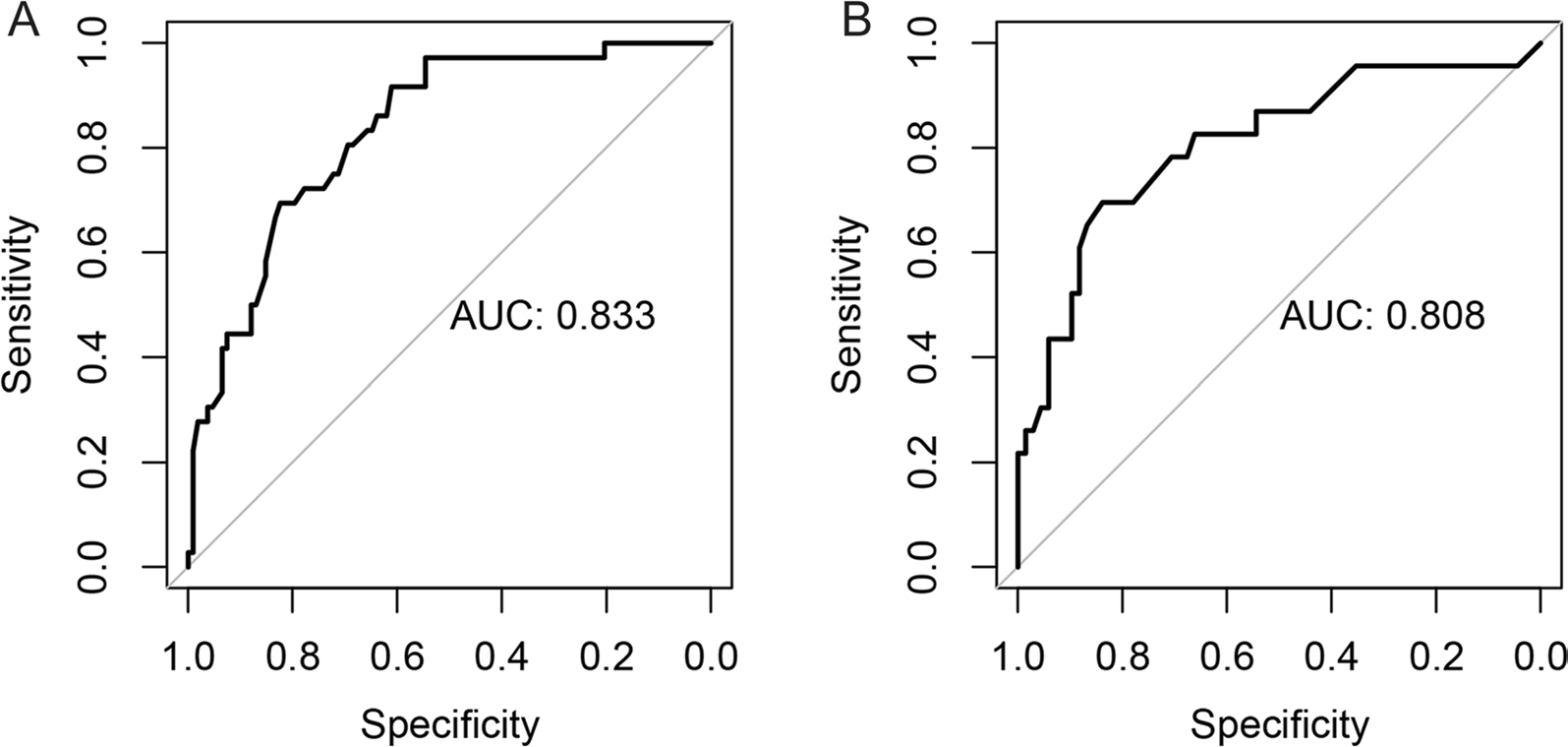
Receiver operating characteristic (ROC) curve of the prediction model in the training (**A**) and validation (**B**) sets

**Fig. 4:**
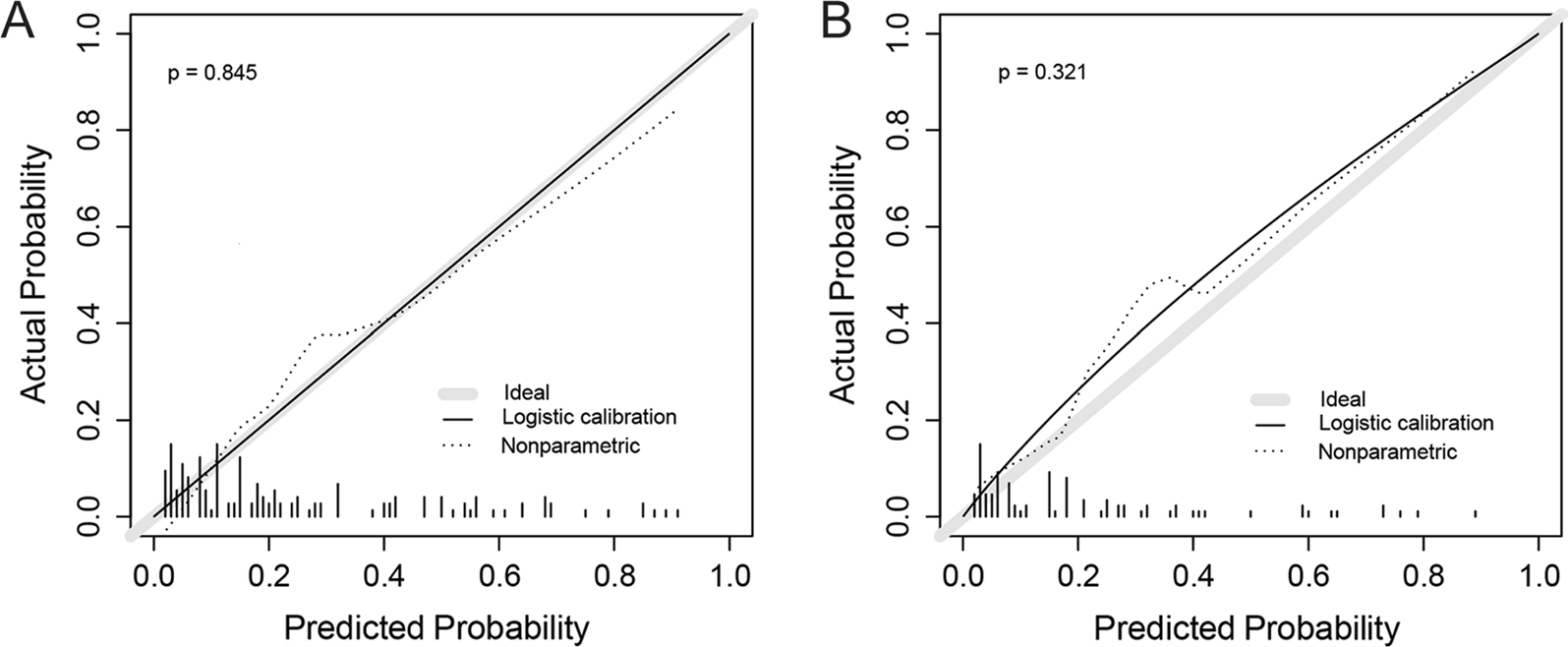
Calibration curves of the nomogram in the training (**A**) and validation (**B**) sets. The x axis represents nomogram prediction. The y axis represents actual probability. The gray line represents a perfect prediction by an ideal model. The solid line represents the bias-corrected performance of the nomogram, where a closer fit to the gray line represents a better prediction

**Fig.5:**
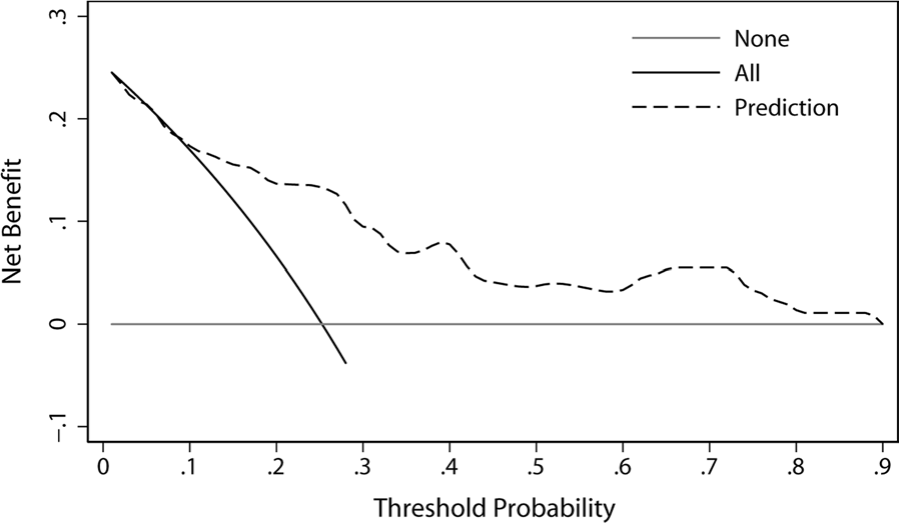
Decision curve analysis (DCA) for the nomogram model. The y axis measures the net benefit. The x-axis represents the threshold probability. The black line represents the hypothesis that all patients have cervical lymph node metastasis. The gray line represents the hypothesis that no patients have cervical lymph node metastasis

A nomogram for the prediction of cervical LN metastasis was constructed with four independent risk factors identified in multivariate analysis (Fig. 2). The prediction model using the training dataset showed good discrimination by using ROC curve analysis. The AUC of the training and validation dataset were 0.833 (95% CI 0.762–0.905) and 0.808 (95% CI 0.696–0.920), respectively (Fig. 3A, B). The calibration curve for the probability of cervical LN metastasis in the training and validation datasets demonstrated good agreement between prediction and observation (Fig. 4A, B). The clinical decision curve of the nomogram was presented in Fig. 5. The results showed that if the risk threshold of a patient was greater than 10%, using the nomogram to predict cervical LN metastases adds more benefit than either the treat-all or treat-none scheme.

## Discussion

Esophageal cancer is still a lethiferous neoplasm that seriously threatens global human health. The surgical methods are rapidly progressing especially in the choice of the dissection of the LN. However, the range of LN dissection in resectable middle and lower thoracic ESCC remains controversial. Three-field lymphadenectomy has been widely accepted as a standard procedure for ESCC patients in Japan but is not yet the world standard [17, 18]. Cervical LN metastases are common in ESCC patients, with a metastatic rate of at least 20 percent [19]. Unfortunately, until now, there is no optimum preoperative method for predicting the status of cervical LN. Three-field LN dissection may be a good choice for patients with tumor-positive cervical LNs but need more precise indicators to select appropriate patients [20, 21].

In this study, we developed a nomogram to predict the probability of cervical LN metastases in middle and lower thoracic ESCC patients. A total of 4 variables were included in the final predictive model. The maximum diameter of tumor and CT-reported cervical LN status could be easily obtained from preoperative routine examinations. The PLN and RLN LN status could be obtained by intraoperative frozen sections. Therefore, this model is convenient in clinical application. Our nomogram also showed reliable clinical performance by the examination of the ROC curve, calibration curve, and DCA curve. Using the nomogram, patients with high scores (> 150 points) are 80% more likely to develop cervical LN metastases.

Tumor length was an important prognostic predictor for ESCC and even was recommended to be incorporated into the TNM staging system [22–24]. Longer tumor length reflected worse biological behavior, which might also indicate a higher probability of LN metastases. Haisley et al. [25] found that each 1-cm increase in esophageal tumor length increased the odds of having positive nodes 3.55 times. Meanwhile, in Rice et al.’s [26] study, longer tumor length was also proved to be a strong predictor of LN metastases in esophageal cancer. Furthermore, Chen et al. [27] reported that tumor length was a risk factor of cervical LN metastases. Biologically, it seems reasonable that longer tumor length in the lymphatic-rich esophageal submucosa may be a more important factor resulting in lymph node metastasis than tumor depth in a single area [25]. In this study, our results revealed that the maximum diameter of tumor was associated with cervical LN metastases.

Lymphatic metastasis is an important metastatic pathway in ESCC patients [28]. Once invaded to the submucosa, esophageal tumor cells can be transferred along with the longitudinal lymphatic networks, with the possibility of bi-directional or skip node spread [29, 30]. For patients with ESCC, the RLN LN is one of the most frequently affected sites and a strong predictor of poor prognosis [31]. Wu et al. [32] reported that the median survival time of patients with RLN LN metastasis was 24 months, which was significantly lower than that of patients with no RLN LN metastasis (83 months). Anatomically, the RLN LNs are located at the junction of the neck and chest, leading many authors to hypothesize that the RLN LNs may be the sentinel lymph nodes for cervical LN metastases [33, 34]. Li et al. [35] reported that when the tumor was located in the middle and lower of the esophagus, the metastatic rate of cervical LN was 50.8% in patients with RLN LN metastasis, while the rate was only 28.2% in patients without RLN LN metastasis. Xu et al. [36] also reported that intraoperative pathological examination of RLN LN using frozen sections could predict cervical LN metastasis. In our study, The PLN and RLN LN status were proved to be independent predictors of cervical LN metastasis.

Currently, there are many imaging methods for evaluating the status of cervical LN, including ultrasound, CT scan, magnetic resonance imaging (MRI), and positron emission tomography-computed tomography (PET/CT). Ultrasound is usually the first imaging technique in the assessment of cervical LN, but it is operator‐dependent, with the possibility of missing subtle signs [37]. CT scan examination is a widely applied method for assessing ESCC and cervical LN status [38]. This technique has several advantages: convenience, noninvasiveness, inexpensiveness, and a shorter scanning time. Previous studies reported that the sensitivity of CT evaluation was 30–64.7% [39, 40]. MRI has a better performance in discriminating benign or malignant LNs, but it is a relatively expensive and time-consuming procedure [41]. PET/CT could provide additional metabolic and functional information, but it still remained controversial in evaluating cervical LN status in ESCC [42]. Furthermore, the expensive cost of PET/CT limited its widespread application in practice. In this study, CT was shown to be a reliable tool to predict cervical LN metastasis.

This study has several limitations. First, it was a retrospective study in which the patients were from a single-center, hence there was a potential for selection bias. Second, the number of included patients was relatively small. Finally, this new model was only performed internal validation, larger multicentered external validation studies are still needed to verify the efficacy of the prediction model.

## Conclusion

We developed and validated a nomogram that predicts cervical LN metastasis in patients with middle and lower thoracic ESCC. This nomogram may be useful for helping clinicians make a wise choice in the extent of lymph node dissection.

## Supplementary Information

**Additional file 1.** Lists all the extracted data which were used to generate all the results of this study.

**Additional file 2: Fig. S1.** Schematic diagram of cervical lymph node station. (A) anterior view; (B) right side view. Station 1, cervical paraesophageal lymph nodes; Station 2, deep cervical lymph nodes; Station 3, supraclavicular LNs.

**Additional file 3: Fig. S2.** Schematic diagram of thoracic lymph node station. (A) anterior view; (B) right side view. Station C201, right recurrent laryngeal nerve nodes (lymph nodes and adipose tissue around the right recurrent laryngeal nerve between the beginning of the right vagus nerve reentry and the end of the right subclavian artery); Station C202, left recurrent laryngeal nerve nodes (lymph nodes and adipose tissue around the left recurrent laryngeal nerve on the upper border of the aortic arch and the upper 1/3 left border of the trachea); Station C203, upper thoracic paraesophageal lymph nodes (Anterior and posterior tracheal lymph nodes from the apex of the lung to the inferior border of the azygos vein arch); Station C204, right thoracic paratracheal lymph nodes (lymph nodes on the right side of the trachea between right vagus nerve and paraesophagus); Station C205, subcarinal lymph nodes; Station C206, middle thoracic paraesophageal lymph nodes (lymph nodes around the esophagus between the trachea bifurcation and the inferior border of the inferior pulmonary vein); Station C207, lower thoracic paraesophageal lymph nodes (lymph nodes around the esophagus between the inferior border of the inferior pulmonary vein and the esophagogastric junction); Station C208, inferior pulmonary ligament lymph nodes (lymph nodes within the inferior pulmonary ligament and close to the inferior border of the right inferior pulmonary vein); Station C209, paradiaphragmatic lymph nodes (lymph node on the right cardiophrenic). Station C201 and C202 were defined as recurrent laryngeal nerve lymph node (RLN LN); Station C203, C206, and C207 were defined as paraesophageal lymph node (PLN); Station C204, C205, C208, and C209 were defined as mediastinal lymph node (MLN).

## Data Availability

The dataset generated and analyzed during the current study is available in Additional file 1.

## Abbreviations

LN: Lymph node
ESCC: Esophageal squamous cell carcinoma
EAC: Esophageal adenocarcinoma
PLN: Paraesophageal lymph node
MLN: Mediastinal lymph node
RLN LN: Recurrent laryngeal nerve lymph node
ALN: Abdominal lymph node
ROC: Receiver operating characteristic
AUC: Area under curve
DCA: Decision curve analysis
MRI: Magnetic resonance imaging
PET/CT: Positron emission tomography-computed tomography
