# Supplementary figures and images for: Development and validation of a nomogram for prediction of cervical lymph node metastasis in middle and lower thoracic esophageal squamous cell carcinoma
Source: BMC Gastroenterol. 2022 Apr 3;22:163. doi: 10.1186/s12876-022-02243-8 (PMC8978436; doi:10.1186/s12876-022-02243-8)

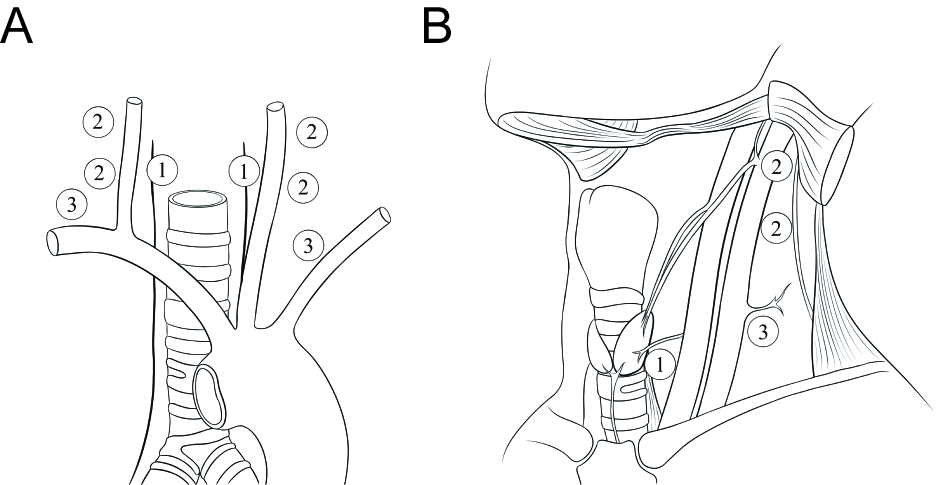

Supplement: Supplementary file 2 — Additional file 2: Fig. S1. Schematic diagram of cervical lymph node station. (A) anterior view; (B) right side view. Station 1, cervical paraesophageal lymph nodes; Station 2, deep cervical lymph nodes; Station 3, supraclavicular LNs. [file 12876_2022_2243_MOESM2_ESM.tif]

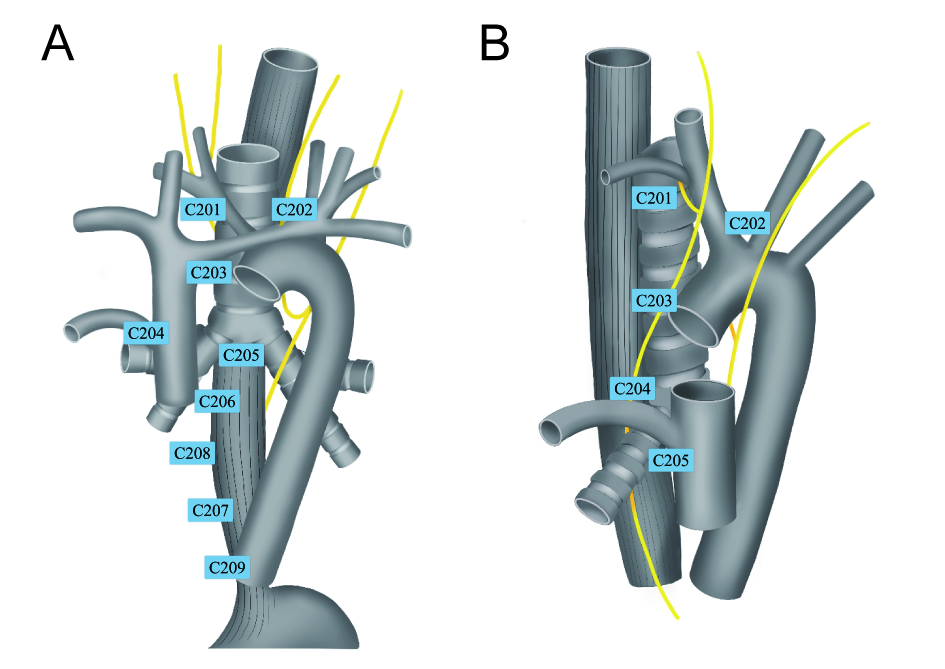

Supplement: Supplementary file 3 — Additional file 3: Fig. S2. Schematic diagram of thoracic lymph node station. (A) anterior view; (B) right side view. Station C201, right recurrent laryngeal nerve nodes (lymph nodes and adipose tissue around the right recurrent laryngeal nerve between the beginning of the right vagus nerve reentry and the end of the right subclavian artery); Station C202, left recurrent laryngeal nerve nodes (lymph nodes and adipose tissue around the left recurrent laryngeal nerve on the upper border of the aortic arch and the upper 1/3 left border of the trachea); Station C203, upper thoracic paraesophageal lymph nodes (Anterior and posterior tracheal lymph nodes from the apex of the lung to the inferior border of the azygos vein arch); Station C204, right thoracic paratracheal lymph nodes (lymph nodes on the right side of the trachea between right vagus nerve and paraesophagus); Station C205, subcarinal lymph nodes; Station C206, middle thoracic paraesophageal lymph nodes (lymph nodes around the esophagus between the trachea bifurcation and the inferior border of the inferior pulmonary vein); Station C207, lower thoracic paraesophageal lymph nodes (lymph nodes around the esophagus between the inferior border of the inferior pulmonary vein and the esophagogastric junction); Station C208, inferior pulmonary ligament lymph nodes (lymph nodes within the inferior pulmonary ligament and close to the inferior border of the right inferior pulmonary vein); Station C209, paradiaphragmatic lymph nodes (lymph node on the right cardiophrenic). Station C201 and C202 were defined as recurrent laryngeal nerve lymph node (RLN LN); Station C203, C206, and C207 were defined as paraesophageal lymph node (PLN); Station C204, C205, C208, and C209 were defined as mediastinal lymph node (MLN). [file 12876_2022_2243_MOESM3_ESM.tif]
